# What the papers say

**DOI:** 10.1093/jhps/hny014

**Published:** 2018-05-16

**Authors:** Ajay Malviya

**Affiliations:** Consultant Orthopaedic Surgeon - Northumbria Healthcare NHS Foundation Trust, Senior Lecturer, Regenerative Medicine - ICM, Newcastle University, 10 East Brunton Wynd, Newcastle upon Tyne, NE13 7BR, UK

The *Journal of Hip Preservation Surgery* (*JHPS*) is not the only place where work in the field of hip preservation may be published. Although our aim is to offer the best of the best, we continue to be fascinated by work that finds its way into journals other than our own. There is much to learn from it, so *JHPS* has selected 6 recent and topical subjects for those who seek a summary of what is taking place in our ever-fascinating world of hip preservation. What you see here are the mildly edited abstracts of the original articles to give them what *JHPS* hopes is a more readable feel. If you are pushed for time, what follows should take you no more than 10 minutes to read. So here goes …

## RANDOMIZED CONTROLLED TRIAL (RCT) COMPARING ARTHROSCOPIC SURGERY AND PHYSIOTHERAPY FOR FEMOROACETABULAR IMPINGEMENT

While the results of 2 multicentred trials (FASHIoN and FAIT), both based in United Kingdom, have been recently presented and we are waiting to see them in print form, we now have the first published RCT on this topic. The results however are not reflecting what the other published evidence suggests.

Mansell *et al*. [1] aimed to determine the comparative effectiveness of surgery and physical therapy for femoroacetabular impingement syndrome in an RCT. Patients were recruited from a large military hospital; of 104 eligible patients, 80 elected to participate, and the majority (91.3%) were active-duty service members. No patients withdrew because of adverse events. The authors randomly selected patients to undergo either arthroscopic hip surgery (surgery group) or physical therapy (rehabilitation group). Patients in the rehabilitation group began a 12-session supervised clinic program within 3 weeks, and patients in the surgery group were scheduled for the next available surgery at a mean of 4 months after enrollment. Patient-reported outcomes of pain, disability and perception of improvement over a 2-year period were collected. The primary outcome was the Hip Outcome Score (HOS; 2 subscales: activities of daily living and sport). Secondary measures included the International Hip Outcome Tool (iHOT-33), Global Rating of Change (GRC) and return to work at 2 years. The primary analysis was on patients within their original randomization group.

Statistically significant improvements were seen in both the groups on the HOS and iHOT-33, but the mean difference was not significant between the groups at 2 years (HOS activities of daily living, 3.8; HOS sport, 1.8; iHOT: 33 and 6.3). The median GRC across all patients was that they “felt about the same” (GRC = 0). Two patients assigned to the surgery group did not undergo surgery, and 28 patients in the rehabilitation group ended up undergoing surgery. A sensitivity analysis of “actual surgery” to “no surgery” did not change the outcome. Twenty (33.3%) patients who underwent surgery and 4 (33.3%) who did not undergo surgery were medically separated from military service at 2 years.

The authors concluded that at 2 years there was no significant difference between the groups, and most patients perceived little to no change in status and one-third of military patients were not medically fit for duty. The authors acknowledged the limitations including a single hospital, a single surgeon and a high rate of crossover; all of these are up for debate until the results of the other RCTs are published.

## EFFECTS OF ARTHROSCOPY FOR FEMOROACETABULAR IMPINGEMENT (FAI) SYNDROME ON QUALITY OF LIFE AND ECONOMIC OUTCOMES

One of the primary questions asked by health-care providers is the cost-effectiveness of hip arthroscopic intervention in terms of quality of life. Mather *et al*. [2] examined the societal and economic impact of hip arthroscopy by high-volume surgeons for patients with FAI syndrome aged <50 years with noncontroversial diagnosis and indications for surgery.

The cost-effectiveness of hip arthroscopy versus nonoperative treatment was evaluated by calculating direct and indirect treatment costs. Direct cost was calculated with Current Procedural Terminology medical codes associated with FAI treatment. Indirect cost was measured with the patient-reported data of 102 patients who underwent arthroscopy and from the reimbursement records of 32 143 individuals between the ages of 16 and 79 years who had information in a private insurance claims data set. The indirect economic benefits of hip arthroscopy were inferred through regression analysis to estimate the statistical relationship between functional status and productivity. A simulation-based approach was then used to estimate the change in productivity associated with the change in functional status observed in the treatment cohort between baseline and follow-up. To analyze cost-effectiveness, 1-, 2- and 3-way sensitivity analyses were performed on all variables in the model, and Monte Carlo analysis evaluated the impact of uncertainty in the model assumptions.

Analysis of indirect costs identified a statistically significant increase in mean aggregate productivity of $8968 after surgery. Cost-effectiveness analysis showed a mean cumulative total 10-year societal savings of $67 418 per patient from hip arthroscopy versus nonoperative treatment. Hip arthroscopy also conferred a gain of 2.03 quality-adjusted life-years over this period. The mean cost for hip arthroscopy was estimated at $23 120 ± $10 279, and the mean cost of nonoperative treatment was estimated at $91 602 ± $14 675. In 99% of the trials, hip arthroscopy was recognized as the preferred cost-effective strategy.

The work has demonstrated that FAI syndrome produces a substantial economic burden on the society which may be reduced through the indirect cost savings and economic benefits from hip arthroscopy.

## DOES FORMAL CAPSULAR REPAIR AT THE TIME OF HIP ARTHROSCOPY IMPROVE CAPSULAR HEALING?

Capsular repair at the time of hip arthroscopy is a matter of debate, and it adds time to the procedure, additional cost in terms of equipment used, is technically challenging and indeed it is not clear whether it improves the outcome. In a multicentred randomized controlled collaboration between centers in New Zealand and United States, the authors[3] have tried to evaluate the magnetic resonance imaging (MRI) appearance of the hip capsule in patients with femoroacetabular impingement (FAI) who underwent simultaneous bilateral hip arthroscopy through an interportal capsulotomy with each hip randomized to undergo capsular repair or not undergo such a repair.

This double-blind, randomized controlled trial included 15 patients (30 hips), with a mean age of 29.2 years, who underwent simultaneous bilateral hip arthroscopy utilizing a small (<3 cm) interportal capsulotomy for the treatment of FAI. The first hip treated in each patient was intraoperatively randomized to undergo capsular repair or no capsular repair. The contralateral hip then received the opposite treatment. MRI was performed at 6 and 24 weeks postoperatively, and the scans were analyzed by 2 musculoskeletal radiologists.

The patients and the radiologists were blinded to the treatment performed on each hip. Capsular dimensions were measured at the level of the healing capsulotomy site and, for hips with a persistent defect, at locations both proximal and distal to the defect. These values were then analyzed at both time points to assess the rate and the extent of capsular healing.

At 6 weeks postoperatively, a continuous hip capsule (with no apparent capsulotomy defect) was observed in 8 hips treated with capsular repair and 3 hips without such a repair. Of the 19 hips with a discontinuous capsule at 6 weeks, 17 were available for follow-up at 24 weeks postoperatively; all 17 demonstrated progression to healing, with a contiguous appearance without defects and no difference in capsular dimensions between the treatment cohorts.

The authors concluded that arthroscopic repair of a small interportal hip capsulotomy site yields an insignificant increase in the percentage of continuous hip capsules seen on MRI at 6 weeks postoperatively compared with no repair. Repaired and unrepaired capsulotomy sites progressed to healing with a contiguous appearance on MRI by 24 weeks postoperatively.

## ROLE OF PLATELET-RICH PLASMA (PRP) in GREATER TROCHANTERIC PAIN SYNDROME—RESULTS OF A RANDOMIZED CONTROLLED TRIAL

Continuing with the theme of generating high-level evidence, Fitzpatrick *et al*. [4] from Australia have looked at the role of PRP injection in comparison with corticosteroid for gluteal tendinopathy.

There were 228 consecutive patients referred with gluteal tendinopathy who were screened to enroll 80 participants; 148 were excluded for various reasons. Participants were randomized (1:1) to receive either a blinded glucocorticoid or an PRP injection intratendinously under ultrasound guidance. A pain and functional assessment was performed using the modified Harris hip score (mHHS) at 0, 2, 6 and 12 weeks and the patient acceptable symptom state (PASS) and minimal clinically important difference (MCID) at 12 weeks.

Participants had a mean age of 60 years, a ratio of female to male of 9:1 and a mean duration of symptoms of >14 months. Pain and function measured by the mean mHHS showed no difference at 2 weeks (corticosteroid: 66.95 versus PRP: 65.23) or 6 weeks (corticosteroid: 69.51 versus PRP: 68.79). The mean mHHS was significantly improved (*P* = 0.048) at 12 weeks in the PRP group (74.05) compared with the corticosteroid group (67.13). The proportion of participants who achieved an outcome score of ≥74 at 12 weeks was 17 (45.9%) of 37 in the corticosteroid group and 25 (64.1%) of 39 in the PRP group. The proportion of participants who achieved the MCID of more than 8 points at 12 weeks was 21 (56.7%) of 37 in the corticosteroid group and 32 (82%) of 39 in the PRP group (*P* = 0.016).

The authors concluded that patients with chronic gluteal tendinopathy >4 months, diagnosed with both clinical and radiological examinations, achieved greater clinical improvement at 12 weeks when treated with a single PRP injection than those treated with a single corticosteroid injection.

## INTERMEDIATE-TERM HIP SURVIVORSHIP AND PATIENT-REPORTED OUTCOMES OF PERIACETABULAR OSTEOTOMY: THE WASHINGTON UNIVERSITY EXPERIENCE

The Bernese periacetabular osteotomy (PAO) has been an alternative to arthroplasty for treating symptomatic acetabular dysplasia, but there have been few studies on the intermediate-term outcomes of this procedure. In this study, the authors assessed intermediate-term hip survival and patient-reported outcomes of PAO [5].

From July 1994 to August 2008, 238 hips (206 patients) were treated with PAO. Sixty-two had a diagnosis other than classic acetabular dysplasia, and 22 were lost to follow-up. The remaining 154 hips (129 patients) were evaluated at an average of 10.3 years postoperatively. Kaplan–Meier analysis was used to assess survivorship with an end point of total hip arthroplasty (THA). Hips were evaluated using the University of California at Los Angeles (UCLA) Activity Score, modified Harris hip score (mHHS) and Western Ontario and McMaster Universities Osteoarthritis Index (WOMAC) pain subscale score. A WOMAC pain subscale score of  ≥10 and/or an mHHS of  ≤70 was considered to indicate a clinically symptomatic hip.

Kaplan–Meier analysis revealed a hip survival rate of 92% at 15 years postoperatively. Eight (5%) hips underwent THA at a mean of 6.8 years. Twenty-four (16%) additional hips were considered symptomatic based on a WOMAC pain score of  ≥10 and/or an mHHS of  ≤70. One hundred and twenty-two (79%) hips did not undergo THA and did not meet the criteria for symptoms, and these hips had a mean mHHS of 92.4, WOMAC pain subscale score of 1.2 and UCLA Activity Score of 7.7 at a mean of 10.1 years. A higher risk of failure was associated with fair or poor preoperative joint congruency (odds ratio [OR]: 8.65; *P* = 0.034) and with a postoperative lateral center-edge angle of  >38° (OR: 8.04). A concurrent head–neck osteochondroplasty was associated with a decreased risk of failure (OR: 0.27; *P* = 0.016).

This study demonstrates the durability of the Bernese PAO. Fair or poor preoperative joint congruency and excessive postoperative femoral head coverage were found to be predictors of failure, while concurrent head–neck osteochondroplasty in patients with an inadequate range of motion after PAO was associated with a decreased risk of failure.

## PREVIOUS HIP PRESERVING SURGERY ADVERSELY AFFECTS THE OUTCOME OF TOTAL HIP ARTHROPLASTY (THA)

This subject has been explored in 2 recent studies; Osawa *et al*. [6] from Nagoya, Japan, have looked at outcome of THA after periacetabular osteotomy (PAO) and Konopka *et al*, [7] from NY, USA, have looked at the same after hip arthroscopic surgery.

Osawa *et al*. [6] performed a case–control study of 27 (29 hips) patients who underwent THA after PAO (osteotomy group); their mean age at surgery was 57.2 years, and they underwent postoperative follow-up for a mean period of 3 years.

For the control group, after matching age, sex and Crowe classification, they included 54 (58 joints) patients who underwent primary THA for hip dysplasia.The 2 groups demonstrated no significant difference in the preoperative Harris hip score, each domain of the SF-36, JHEQ and the VAS score of hip pain and satisfaction. The osteotomy group demonstrated significantly poor Harris hip scores for gait and activity and JHEQ for movement at the last follow-up. There was no significant difference in each domain of the SF-36 and the VAS score of hip pain and satisfaction at the last follow-up. They concluded that previous PAO affects the quality of physical function in patients who undergo subsequent THA.

Konopka *et al*. [7] in a cohort of 5091 patients who underwent hip arthroscopy, identified 69 patients who underwent subsequent THA (46) or hip resurfacing arthroplasty (23). Patients were matched to patients with no history of hip arthroscopy. Patients who underwent THA with history of arthroscopy had lower postoperative hip disability and Osteoarthritis Outcome Score Pain (82 versus 93, *P* = 0.003), stiffness (85 versus 93, *P* = 0.01), sports and recreation (71 versus 88, *P* = 0.003), quality of life (65 versus 86 ±, *P* < 0.0001), WOMAC Pain (86 versus 93, *P* = 0.03), WOMAC stiffness (80 versus 88, *P* = 0.05) and Short Form-12 Physical Component Scores (48 versus 54, *P* = 0.008). They were less likely to be “very satisfied” after arthroplasty (71% versus 89%, *P* = 0.0008).

It seems that both PAO and hip arthroscopy before hip arthroplasty is associated with slightly poorer results in several patient-reported outcomes. These results are relevant when consenting these patients for arthroplasty.
